# Association of knowledge on ART line of treatment, scarcity of treatment options and adherence

**DOI:** 10.1186/s12913-016-1483-6

**Published:** 2016-07-15

**Authors:** Habib O. Ramadhani, Charles Muiruri, Venance P. Maro, Michael Omondi, Julian B. Mushi, Eileen S. Lirhunde, John A. Bartlett

**Affiliations:** Kilimanjaro Christian Medical Centre (KCMC), P.O Box 3010 Moshi, Kilimanjaro Tanzania; Kilimanjaro Christian Medical University College, Moshi, Tanzania; Division of Infectious Diseases and International Health, Department of Medicine, Duke University Medical Center, Durham, NC USA; Duke Global health Institute, Durham, NC USA; Kilema Designated District Hospital, Moshi, Tanzania; Kibosho Designated District Hospital, Moshi, Tanzania

**Keywords:** HIV, Anti-retro-viral, Adherence, Knowledge of ART line of treatment

## Abstract

**Background:**

Adherence to Antiretroviral Therapy (ART) is critical piece in the management of HIV infected patients. Despite the benefits of ART, non-adherence to ART persists. This study explores association between patient’s knowledge of the ART line of treatment, availability of future treatment options and adherence.

**Methods:**

A cross sectional survey of HIV infected adolescent and adults was conducted. Cumulative optimal and sub-optimal adherence was defined as percentage adherence of ≥ 95 % and < 95 %, respectively. Binomial regression models were used to assess the association of patient’s knowledge of the ART line of treatment, availability of future treatment options and adherence.

**Results:**

Of the 402 patients reviewed, 101 (25.1 %) patients knew their ART line of treatment and were aware that future treatment options are limited. Compared to those who were not aware of the ART line of treatment and/or scarcity of future treatment options, those who were aware were more likely to be adherent (adjusted prevalence ratio [APR], 1.1; 95 % CI, 1.0–1.3).

**Conclusion:**

The study reports knowledge of patient’s ART line of treatment and future treatment options is important indicator of adherence to ART. Although majority of the patients did not have the knowledge, those who had the knowledge demonstrated to be more adherent. It is critical for the physicians/health care providers in these settings to clearly educate patients about ART line of treatment and limited availability of future treatment options as such information is likely to influence individual behavior and improve patient’s adherence to ART.

## Background

Adherence to antiretroviral therapy (ART) is essential for the successful management of HIV-infected patients. Clinical trials and observational studies have repeatedly shown that patients taking their prescribed medications as recommended are likely to be virologically suppressed [1–3]. Despite the benefits of ART and the known need to adhere, non-adherence to ART persists. Recent findings indicate that patients who were non-adherent to first line ART continued to be non-adherent to second-line ART, suggesting that ART non-adherence is still a major concern in the management of HIV-infected patients [4]. Furthermore, in most resource-limited settings only two lines of ART are available. Advances in ART-based HIV treatment and preventive strategies such as “Treatment as Prevention” (TasP), including prevention of mother to child transmission (PMTCT) and pre-exposure prophylaxis (PreP), have shown promising results in preventing HIV transmission. For example, treatment of the HIV-infected member in sero-discordant couple reduced the risk of new HIV infections in the un-infected partner [5]; however, the success of such strategies requires high degree of medication adherence. To be virally suppressed, at least 95 % adherence is required for a number of combined ART regimens [6] and for those containing boosted protease inhibitors, adherence of at least 80 % is warranted [7, 8].

Barriers to adherence with ART have been explored [9–11]; however, the association between a patient’s knowledge of the ART line of treatment, awareness of the availability of future treatment options, and adherence to ART is lacking. Although non-adherence to ART medications may be primarily considered a personal behavioral problem, continued support with clear understanding of ART line of treatment as well as patient awareness of the limited availability of treatment options may be beneficial in improving patient ART adherence. For example, patient awareness of the limited availability of second-line ART following first-line ART failure may modify individual behavior and hence improve adherence. Due to the expenses associated with second-line ART as well as limited availability of future treatment options among patients failing first-line ART, we sought to explore the association between a patient’s knowledge of the ART line of treatment, the availability of future treatment options, and adherence. In most resource limited settings including Tanzania, second-line ART is the final salvage regimen following first line failure. It is not known if knowledge of ART line of treatment and awareness of the limited availability of future treatment options may be associated adherence to ART. Whether patients who have the knowledge of the ART line of treatment and are aware that future treatment options are limited are likely to be more adherent than those without this knowledge it is not understood. Exploring association between knowledge of limited treatment options and adherence is critical because such a finding will add to the existing body of knowledge of factors associated with adherence, and potential interventions that improve patient adherence with ART. We explored this association using both cross-sectional and longitudinally collected observational data from five care and treatment centers (CTC) located within Kilimanjaro Region in Northern Tanzania.

## Methods

### Study design and population

We used a cross-sectional study design to evaluate the association of knowledge of ART line of treatment, and the limited availability of future ART treatment options, with adherence to first-line ART. The study population included HIV-infected adolescent and adult patients attending CTCs at the Kilimanjaro Christian Medical Center (KCMC), Mawenzi Regional Hospital (MRH), Kibosho, and Kilema District Hospitals, and Majengo Health Center in Northern Tanzania between January 2004 and May 2015. These CTCs offer treatment according to the Tanzanian Ministry of Health treatment guidelines for the provision of ART. This is a secondary data analysis involving participants who are part of the primary study that explores predictors of viral suppression among HIV-infected patients in those clinics. Only participants who have been on treatment for at least 2 years in the primary data were included.

### Data collection

After obtaining informed consent, standardized questionnaires translated into Kiswahili were administered to participating patients by trained research nurses. Among other things, questionnaire addressed demographic characteristics, knowledge of ART line of treatment, and availability of future treatment options.

As part of routine HIV clinical care in Tanzania, all patient data including demographics, medication use, adherence indicators [good adherence (G) = fewer than 2 missed days per month/poor adherence (P) = 2 or more missed days per month], were collected on standardized forms by health care providers in those clinics and entered into the National database. These standardized forms were reviewed for an adherence assessment, and all patients with adherence information for at least 24 months from the last clinic visit were included. Recognizing adherence wanes over time cumulative adherence rating was used [12, 13]. For this study, percentage adherence was then recalculated using 24 months of observations as the common denominator, adherence = [number of recorded G’s/(P + G)]* 100. For example, a person with 22 recorded G’s and two recorded P’s has an adherence of 91.7 %. The resulting adherence percentage was later dichotomized as optimal (adherence ≥ 95 %) or sub-optimal (adherence < 95 %) based on previous findings [6]. A near perfect adherence defined by adherence ≥ 95 % is widely used for sustained viral suppression [14]. Cumulative adherence assessment has been used by other studies [4, 15].

### Definition of variables

The primary endpoint was the cumulative percentage adherence to first-line ART, as defined above. The exposure of interest was the participant’s knowledge of ART line of treatment, and the availability of future treatment options. Patients were asked series of questions such as “which line of treatment are you on?”, “can you mention other line of treatment?” and “have you been told that apparently second-line is the final regimen at your clinic?” Using this information we defined knowledge on ART = 1 if participants correctly stated his/her ART line of treatment (first-line or second-line) and were aware that future treatment options were limited. Knowledge on ART was scored as zero if participants did not know their ART line of treatment and/or were not aware that future treatment options were limited. We verified the ART line of treatment reported by patients through a review of their medical records. Other variables were evaluated including age, gender, duration on first-line ART, treatment sites, marital status, education, plasma HIV RNA levels (if available), and CD4 cell count at the time of interview.

### Statistical analyses

Statistical analyses were conducted using SAS version 9.3 (SAS Institute Inc., Cary, NC). Frequencies of categorical variables were calculated as the proportions of patients sampled. Crude and adjusted binomial regression models were used to assess the association of a patient’s knowledge of ART line of treatment, availability of future treatment options and adherence. All associations were presented as adjusted prevalence ratios (APRs) with 95 % confidence intervals (CIs). Estimates whose CI crossed 1 were considered non-statistically significant. All other variables were added into the model and backward elimination was done with the final model having variables whose change in estimate was ≥ 10 %.

## Results

We surveyed a sample of 450 patients who were on ART between January 2004 and May 2014. Of these, 402 (89.3 %) were on first-line ART and were used for final analysis. Most were female (271, 67 %), and the majority were between 30 and 55 years old (301; 75 %); (Table 1). The greatest proportion of patients came from Mawenzi Regional Hospital (223; 53 %). Most patients were not aware that they were on first-line ART (321; 80 %), and were unaware that future treatment options were limited (317; 79 %). Approximately half of patients had been on first-line ART for longer than 5 years (199; 50 %). The majority had less than primary school education (332; 83 %), 199 (49.5 %), and 183 (45.5 %) were either divorced/separated or widowed. The latest CD4+ cell count at the time of interview was less than 350 c/mm^3^ for 224 (56 %) participants, and 252 (56.0 %) had HIV RNA less than 1000c/ml.

**Table 1:** Demographic and Clinical Characteristics of HIV-Infected Patients Receiving Care at Five Care and Treatment Centers in Kilimanjaro Region, Moshi, Tanzania 2004–2015

Characteristics	All Patients *n* = 402	Optimal Adherence *n* = 223	Sub-optimal Adherence *n* = 179
Age			
< 30	19 (4.7)	14 (6.3)	5 (2.8)
30–55	301 (74.9)	169 (75.8)	132 (73.7)
> 55	82 (20.4)	40 (17.9)	42 (23.5)
Gender			
Male	131 (32.9)	87 (39.0)	44 (24.6)
Female	271 (67.1)	136 (61.0)	134 (75.4)
Education			
≤ primary school education	332 (82.6)	188 (84.3)	144 (80.4)
> primary school education	70 (17.4)	35 (15.7)	35 (19.6)
Marital status			
Not married	57 (14.1)	33 (14.8)	24 (13.4)
Married	162 (40.3)	92 (41.3)	80 (39.1)
Divorced/Separated/Widowed	183 (45.5)	98 (43.9)	75 (47.5)
CD4 at the time of interview			
< 350 cells/mm^3^	224 (55.7)	129 (57.9)	95 (53.1)
≥ 350 cells/mm^3^	178 (44.3)	94 (42.1)	84 (46.9)
Duration on treatment			
< 36 months	130 (32.3)	67 (30.0)	63 (35.2)
36–60 months	73 (18.2)	49 (22.0)	24 (14.4)
> 60 months	199 (49.5)	107 (48.0)	92 (51.4)
Knowledge of ART			
No	301 (74.9)	158 (70.8)	143 (79.9)
Yes	101 (25.1)	65 (29.2)	36 (20.1)
Knows is on first-line ART			
No	321 (79.9)	173 (77.6)	148 (82.7)
Yes	81 (20.1)	50 (22.4)	31 (17.3)
Knows other options are limited			
No	317 (78.9)	168 (75.3)	149 (83.2)
Yes	85 (21.1)	55 (24.7)	30 (16.8)
Plasma HIV RNA level			
< 1000 c/ml	252 (56.0)	88 (44.7)	164 (64.8)
≥ 1000 c/ml	198 (44.0)	109 (55.3)	89 (35.2)
Sites			
KCMC	75 (16.7)	52 (26.4)	23 (9.1)
Mawenzi	223 (49.5)	69 (35.0)	154 (60.9)
Others	152 (33.8)	76 (38.6)	76 (30.0)

*Abbreviations*: *KCMC* Kilimanjaro Christian Medical Center, *N/A* Not applicable, *HIV RNA* Human Immunodeficiency Virus Ribonucleic Acid

### Adherence rating

One hundred and seventy-nine participants (55.5 %) had optimal adherence to ART. After adjusting for age, gender, education, marital status, CD4 cell count, duration on treatment, plasma HIV RNA levels and CTC sites, patients who knew their ART line of treatment and were aware that future treatment options were limited were more likely to have optimal adherence to ART than those who had no knowledge of their ART line of treatment and/or were unaware that future treatment options were limited (prevalence ratio [PR], 1.1; 95 % CI, 1.0–1.3) (Table 2).

**Table 2:** Crude and Adjusted Risk Factors of Optimal Adherence to First-Line ART among HIV-Infected Adults Receiving Care at Five Care and Treatment Centers in Kilimanjaro Region, Moshi, Tanzania 2004–2015

Characteristics	Bivariable Analysis Prevalence Ratio 95 % CI	Multivariable Analysis Prevalence Ratio 95 % CI
Age		
< 30	1	1
30–55	0.8 (0.6–1.0)	0.8 (0.6–1.1)
> 55	0.7 (0.5–0.9)	0.8 (0.6–1.0)
Gender		
Male	1	1
Female	1.3 (1.1–1.6)	1.1 (1.0–1.3)
Education		
≤ primary school education	1	1
> primary school education	0.9 (0.7–1.1)	0.9 (0.8–1.1)
Marital status		
Not married	1	1
Married	1.0 (0.8–1.2)	1.0 (0.8–1.3)
Divorced/Separated/Widowed	0.9 (0.7–1.2)	1.0 (0.8–1.3)
CD4 at the time of interview		
< 350 cells/mm^3^	1	1
≥ 350 cells/mm^3^	0.9 (0.8–1.1)	1.0 (0.9–1.1)
Duration on treatment		
< 36 months	1	1
36–60 months	1.3 (1.0–1.6)	1.2 (1.0–1.3)
> 60 months	1.0 (0.8–1.3)	1.0 (0.9–1.2)
Knowledge of ART		
No	1	1
Yes	1.2 (1.0–1.5)	1.1 (1.0–1.3)
Plasma HIV RNA level		
< 1000 c/ml	1	1
≥ 1000 c/ml	0.6 (0.5–0.7)	0.8 (0.7–0.9)
Sites		
KCMC	1	1
Mawenzi	1.3 (1.0–1.8)	1.2 (1.0–1.4)
Others	1.1 (0.8–1.6)	1.1 (0.9–1.4)

*Abbreviations*: *KCMC* Kilimanjaro Christian Medical Center, *N/A* Not applicable, *HIV RNA* Human Immunodeficiency Virus Ribonucleic Acid

Several other factors were associated with ART adherence with the estimates about the same as that of knowledge on ART. For example, women were more likely (APR, 1.1; (1.0–1.3) than men to have optimal adherence to ART. Compared to patients who have been on treatment for less than 36 months, those who have been on treatment between 36 and 60 months were more likely to have optimal adherence [APR, 1.2; (1.0–1.3)]. CTC site was also positively associated with adherence to ART, and patients from Mawenzi Regional Hospital were more likely to have optimal adherence than those from the KCMC Consultant Hospital (APR, 1.2; (1.0–1.4). Plasma viral load was inversely associated with optimal adherence, as those patients with plasma HIV RNA levels ≥ 1000 c/ml were less likely to have optimal adherence compared to those whose HIV-RNA < 1000 c/ml [APR, 0.8; (0.7–0.9)].

## Discussion

The importance of adherence to ART cannot be overemphasized. The reduction of morbidity and mortality related to HIV/AIDS and associated opportunistic infection among HIV-infected patients is clear among patients taking their medications as prescribed [16, 17]. Interventions to improve adherence to ART are a priority, and numerous interventions have been explored [18, 19]. In this study we demonstrated that knowledge on ART line of treatment as well as awareness of limited availability of future treatment options is an indicator of adherence. Patients who were aware of their ART line of treatment and who were also informed of limited future treatment options were 10–20 % more likely to adhere to their medications than those who were not aware of either. Approximately three-quarters of the study participants were not aware of their ART line of treatment and/or did not know if future ART treatment options are limited, reflecting a general lack of knowledge and perspective on the use of ART regimens.

The main strengths of this study are that, to our knowledge, it is the first study that assesses association between patient knowledge on ART line of treatment, future treatment options and adherence to ART. The association between patient’s awareness of ART line of treatment and adherence has never been explored previously and is a unique feature of this study; however, some studies have shown associations between other components of ART knowledge and adherence. About 80 % of HIV-infected adults in South Africa had poor knowledge of HIV and ART, and poor medication adherence was partly attributed to the poor knowledge [20]. The high proportion of patients with poor knowledge of ART in this South African study is similar to the observations in our study. The lack of knowledge in this setting could be factors at the patient and health care provider level. At the patient level, high level of illiteracy (83 % had equal to or less than primary school education). Failure to attend ART education sessions also explains low level of knowledge. At the provider level, low doctor to patient ratio leads to reduced amount of time that physicians spend with the patients [21] thereby limiting the amount of health education patient receives. In addition, absence of consistent health education or the presence of incompetent health educators who are not fully aware of ART regimens and their availability may explain the lack of this knowledge. Treatment preparation is an important component in the management of HIV patients, however, it may not be conducted across all sites as required and if conducted, it may not be done serially, which further may explain low levels of patient knowledge. Similar cross sectional analyses from African setting reported low level of knowledge about other component of ART some of which were associated with poor adherent. For example,a study done in Togo showed that only 55 % of the HIV-infected patients were aware of the names of their prescribed ART medication (s) [22]. Over 90 % of HIV-infected women in Ghana had inadequate knowledge on ART, and these women were much more likely to default from PMTC care [23]. A study of the association of knowledge on the treatment plan, regimen and adherence was conducted in Ethiopia. About 33 % had knowledge on the treatment plan and regimen, and those who had knowledge were more likely to be adherent than those who did not [24], suggesting knowledge on different components of ART is crucial in improving patient adherence. We have also been able to show gender variations in ART adherence in that woman tended to be more likely than men to adhere to ART medications. A number of studies substantiated this finding [25, 26]. In addition, recognizing adherence wanes over time [12, 13], we used 24-month self-reported patient information to assess cumulative adherence.

Virological outcome was also correlated with adherence among study participants. Patients whose viral loads were more than 1000c/ml were less likely to be adherent than those whose viral loads were less than 1000c/ml, confirming the role of adherence in viral load suppression as evidenced in multiple studies [1–3].

Our study does have a number of limitations. Although we assessed cumulative adherence, it was from self-reported patient information collected by health care providers. Self-reported adherence is subject to over-estimation of adherence percentages. Nevertheless, in this study we were able to correlate optimal adherence with viral load results. When the viral loads were related to patient adherence, those with higher viral loads (≥ 1000c/ml) were less likely to be adherent than those with low values of viral load (< 1000 c/ml). Such a finding suggests that our adherence percentages may have not been overestimated. In addition, self-reported adherence measurement has been extensively used and is associated with clinical, immunological and virological outcomes for patients [11, 27]. In addition, with a cross-sectional study design to assess association between knowledge of ART and adherence, the study design cannot establish a temporal association or causality. Furthermore, because we limited patients who have been on treatment for at least 2 years from the primary data, selection bias is a potential concern. The study might not have captured representative patients to address the research question. However, knowledge of different components of ART has shown to be important indicator of adherence in other studies.

## Conclusion

The study reports patient knowledge of ART line of treatment and future treatment options as an indicator of adherence to ART. Although majority of the patients did not have the knowledge, those who had the knowledge were demonstrated to be more adherent. It is critical for the health care providers in resource-limited settings to clearly educate patients about ART line of treatment and limited availability of future treatment options, and such information may influence individual behavior and improve patient adherence with ART.
